# Work-related neck and upper limb disorders – quantitative exposure–response relationships adjusted for personal characteristics and psychosocial conditions

**DOI:** 10.1186/s12891-019-2491-6

**Published:** 2019-04-01

**Authors:** Istvan Balogh, Inger Arvidsson, Jonas Björk, Gert-Åke Hansson, Kerstina Ohlsson, Staffan Skerfving, Catarina Nordander

**Affiliations:** Division of Occupational and Environmental Medicine, Department of Laboratory Medicine, Lund University, Medicon Village, SE-223 81 Lund, Sweden

**Keywords:** Work-related musculoskeletal disorders, Neck, Shoulder, Wrist, Threshold limit value, Ergonomics, Technical measurements, Inclinometry, Goniometry, Electromyography

## Abstract

**Background:**

We have previously reported quantitative exposure-response relationships between physical exposures recorded by technical methods, and complaints and diagnoses in the neck/shoulders, and the elbows/hands, based on group data. In the present study the number of workers was doubled, and information on individual factors, and psychosocial working conditions was used. Relationships between various kinds of exposure and response have been analysed in this larger and more detailed sample.

**Methods:**

The prevalence of complaints (Nordic Questionnaire) and diagnoses (clinical examination) were recorded in a number of occupational groups within which the participants had similar work tasks, 34 groups of female employees (*N* = 4733 women) and 17 groups of male employees (*N* = 1107 men). Age and other individual characteristics were recorded, as well as psychosocial work environment factors (job-content questionnaire) for most participants. Postures and velocities (inclinometry) of the head (*N* = 505) and right upper arm (*N* = 510), right wrist postures and velocities (electrogoniometry; *N* = 685), and muscular activity (electromyography; EMG) in the right trapezius muscle (*N* = 647) and forearm extensors (*N* = 396) were recorded in representative sub-groups. Exposure-response relationships between physical exposure and musculoskeletal disorders, adjusted for individual factors with Poisson regression were then calculated. The effect of introducing psychosocial conditions into the models was also assessed.

**Results:**

Associations were found between head velocity, trapezius activity, upper arm velocity, forearm extensor activity and wrist posture and velocity, and most neck/shoulder and elbow/hand complaints and diagnoses. Adjustment for age, other individual characteristics and psychosocial work conditions had only a limited effect on these associations. For example, the attributable fraction for tension neck syndrome among female workers with the highest quintile of trapezius activity was 58%, for carpal tunnel syndrome versus wrist velocity it was 92% in men in the highest exposure quintile.

**Conclusions:**

Based on the findings, we propose threshold limit values for upper arm and wrist velocity.

## Background

Musculoskeletal disorders cause major problems in many countries, not only in terms of personal suffering, but also due to the costs associated with loss of production, sickness benefits and health care [1–6]. An increasing number of studies have shown that adverse ergonomic factors at work constitute substantial risks. In particular, awkward postures, high loads, rapid movements, repetitious tasks and lack of adequate recovery time have been identified as harmful, both separately and in combination, to the neck [2, 7–9], shoulders [2, 3, 8–12] and hands [2, 3, 8, 13, 14].

Thus, there is a great need for prevention. Then, to better design sustainable work environments, it is crucial to have sufficiently detailed knowledge on exposure-response relationships, and to use this information to inform development and modification of guidelines for the physical workloads. There are a number of guidelines [15–17]. However, they cover only limited aspects, most often work postures but rarely velocities of work motions.

To determine exposure-response relationships, reliable information regarding exposure is necessary [18], in particular, quantitative measurements of exposure. Information is often collected by self-reporting questionnaires, however, the results obtained in this way may be biased [19]. Observations of the work actually performed are more objective. Although postures can be recorded semi-quantitatively, force and motions are difficult to ascertain [20]. Sometimes, expert judgments on the physical workloads in different occupations have been included in job exposure matrices [21], which may cause observation bias if the expert has a preconception about the exposure [22]. Furthermore, observation over sufficiently long periods of observation for a reliable evaluation requires considerable resources.

Another approach is to apply technical sensors on the body, to assess muscular activity, as well as postures and motions [23, 24]. Such methods provide objective data, which can be collected at high frequency during the whole working day. Finally, to define generic exposure-response relationships, investigations of occupations with a broad range of exposures are necessary, including large groups of workers.

Adequate descriptions of exposure-response relationships are also dependent on reliable records of outcomes. So far, self-administered questionnaire-recorded information has been used most frequently. However, such records run a large risk of reporting-bias affected by the exposure, and are imprecise as to the tissues concerned. Information on which structures that are affected is of interest, as a complement to the workers’ experience of pain or discomfort. To obtain such knowledge, a structured clinical examination should be applied, and diagnoses defined.

Many other factors apart from the physical workload are important in the development of musculoskeletal disorders. Numerous studies have revealed associations between work-related musculoskeletal disorders and the psychosocial/organizational work environment, concerning mainly job demands, control and support. This has been found to be most obvious for neck/shoulder disorders [2, 3, 7, 8, 25], but less clear for elbow/hand disorders [13, 26]. Therefore, it is important to take the psychosocial work environment into account. However, a common problem in the interpretation of the results is the generally close association between reported psychosocial workload and physical risk exposures [27]. This has only occasionally been addressed.

In addition to work-related risk factors, the prevalence of several disorders have been linked to individual risk factors, such as increasing age and high body mass index (BMI), and smoking [7, 28, 29]. Some disorders have also been found to be more prevalent among women than men [7, 8, 28]. Furthermore, circumstances outside work, such as family responsibilities, and the opportunity to exercise or relax and recover, may play a role [30]. Many of these individual risk factors are also associated with both physical and psychosocial workloads, stressing the importance of a detailed covariate assessment in order to improve the reliability of the results of the study.

A number of studies have reported exposure-response relationships [9, 31–41]. However, most of these are based on observations or expert ratings of physical exposure. Current knowledge is thus too limited to establish evidence-based thresholds, at which a workload can be considered harmful. The proposal by ACGIH of a threshold limit value for hand activity was an important advancement [16]; however, some of its components need further refinement [42].

During the past 25 years, we have collected data on physical exposures and self-reported psychosocial work environment and health in numerous occupational groups. The prevalence of neck/shoulder and elbow/hand disorders has been recorded using questionnaires and, in many cases, participants have also undergone a standardized clinical examination, enabling reliable diagnoses to be made. Exposures in terms of head, upper arm and wrist postures and motions, as well as muscular activities in the trapezius and in the forearm extensor muscles have been assessed by technical methods in representative sub-groups [43, 44]. As a result of these studies, we have found several exposure-response relationships, e.g. between the activity of the trapezius muscle and the prevalence of tension neck syndrome [23], and between wrist motions and the prevalence of carpal tunnel syndrome [24]. However, these analyses were performed on a group level, and no adjustments were made for individual risk factors. We then included groups with at least 30 male or female participants [45]. Since then we have examined a large number of workers in several additional occupational groups [46, 47]. We have now synthesized all the data, making it possible to calculate reliable exposure-response relationships, taking into account individual characteristics and the participants’ view of their psychosocial work environment.

The aim of the present study was to investigate the associations between physical workloads in the neck and upper limb, and complaints and diagnoses in these regions, adjusted for age and individual risk factors, using this large data set. We also evaluated the effects of the psychosocial working environment. Attributable fractions in highly exposed participants were estimated for some of the most pronounced associations.

## Methods

### Study design

During the period 1989 to 2013, we collected data on musculoskeletal disorders among workers in many different occupational groups. Technical measurements were made over a wide range of physical exposures. The same methods were used for the collection of data on physical exposure and musculoskeletal disorders throughout this period. Data have also been collected on individual factors, and on the psychosocial work environment in many of the groups using self-reported questionnaires. As the data were collected in a number of different studies, with different designs, the kinds of exposure measured differed, and in some groups, no clinical examination was performed. The data was then assembled in one data-set, allowing calculation of associations between exposure and outcome.

### Participants

In 2009 and 2010 we published the results of studies on occupational musculoskeletal disorders in groups with at least 30 male or female participants [45], and on physical exposure in the neck and upper extremities in representative sub-groups [43, 44]. Since then, we have expanded our database to include 828 female grocery store workers [47], 142 male and 375 female teachers, 925 female nurses and 291 female ultrasound examiners [46]. Furthermore, 236 participants who belonged to 12 occupational groups with less than 30 subjects have also been added. The total database now includes workers from 17 male and 35 female occupational groups in various occupations. The nature of the work tasks ranged from repetitive and/or constrained to varied/mobile.

All the employees at the different workplaces were invited to participate, including those on sick-leave. Eleven participants were excluded as their age was not reported, and the whole database included in the present study thus includes 1107 men and 4733 women.

Table 1 presents the characteristics of all the subjects, i.e. age, time in the present occupation, civil status (married/living with a partner or living alone), BMI, smoking habits, children below 12 years of age living at home, domestic work (> 10 h per week), and exercise (> 1 h per week), obtained by questionnaire or interview.

**Table 1:** Characteristics of all the participants, and the groups of participants categorized by quintiles of assigned right wrist velocity (50th percentile)

	N	All	Wrist velocity (°/s)^a^				
Men			< 4	≥4 – < 8	≥8 – < 17.5	≥17.5 – < 30	≥30
N			232	233	231	219	136
Age (years)	1107	42 (32–51)	45 (35–51)	44 (35–56)	42 (32–51)	36 (27–50)	36 (31–44)
Employment time (years)	1106	10 (4–19)	16 (7–23)	13 (6–26)	10 (3–16)	6 (3–12)	9 (6–17)
BMI (kg/m^2^)	484	25 (23–27)	24 (23–26)	25 (23–27)	25 (23–27)	25 (23–28)	--^b^
Smoker (%)	799	22	17	4	29	32	23
Married/living with partner (%)	887	59	81	84	34	34	81
Young child at home (%)	860	45	49	57	42	30	60
Exercise >once a week (%)	779	51	68	36	62	57	42
Domestic work > 1 h per day (%)	383	46	--^b^	52	49	32	46
Job demands (score 1–4)	859	2.6 (2.2–2.9)	2.6 (2.2–3.0)	2.7 (2.4–3.0)	2.3 (1.8–2.7)	2.6 (2.2–2.9)	2.7 (2.4–2.9)
Job control (score 1–4)	877	2.8 (2.3–3.2)	3.0 (2.7–3.5)	3.1 (2.8–3.3)	3.0 (2.8–3.3)	2.4 (1.9–2.8)	1.9 (1.6–2.3)
Job support (score 1–4)	878	2.9 (2.6–3.2)	3.0 (2.5–3.3)	2.9 (2.6–3.1)	3.0 (2.7–3.3)	2.9 (2.6–3.2)	2.5 (2.3–2.8)
Women			< 6.3	≥6.3 – < 9.9	≥9.9 – < 20	≥20 – < 24.1	≥24.1
N			956	1054	881	813	965
Age (years)	4733	44 (34–53)	45 (36–54)	49 (40–57)	39 (30–50)	40 (30–51)	43 (33–52)
Employment time (years)	4630	11 (5–21)	12 (6–24)	13 (6–28)	10 (4–18)	8 (3–16)	11 (5–20)
BMI (kg/m^2^)	3490	24 (22–26)	23 (21–25)	24 (22–26)	23 (21–26)	24 (22–28)	24 (22–27)
Smoker (%)	3373	22	11	7	44	26	39
Married/living with partner (%)	3930	70	79	81	56	76	51
Young child at home (%)	3957	48	49	55	42	46	43
Exercise >once a week (%)	3402	46	37	33	68	57	53
Domestic work > 1 h per day (%)	2495	60	63	65	49	55	54
Job demands (score 1–4)	3589	2.7 (2.4–3.0)	2.6 (2.3–2.9)	2.9 (2.6–3.1)	2.4 (2.2–2.8)	2.7 (2.4–3.0)	2.7 (2.3–3.1)
Job control (score 1–4)	3613	2.8 (2.4–3.1)	2.8 (2.7–3. 2)	3.0 (2.7–3.3)	2.8 (2 .3–3.2)	2.5 (2.2–2.9)	2.4 (2.0–2.8)
Job support (score 1–4)	3600	2.8 (2.4–3.0)	2.9 (2.6–3.1)	2.9 (2.6 – 3.1)	2.6 (2.3–3.0)	2.8 (2. 4–3.0)	2.3 (2.1–2.8)

*BMI* Body mass index

^a^No data were available on wrist velocity for 56 men and 64 women

^b^No data available

Medians as well as first and third quartile (Q_1_ – Q_3_), or percentages. N denotes the number of participants

### Physical exposure

Participants within each occupational group performed identical or very similar work tasks. Physical exposure was recorded in a sub-sample of workers in each group (usually 10 females or males). All were right-handed, and were unaffected by pain, i.e. they were able to work normally. In each of these worker we most often simultaneously recorded work postures and velocities in head, upper arm and wrist, as well as muscular activity in the trapezius muscle and the forearm extensors, on the right side. In some groups not all methods were used. Recordings were made on one worker at a time, thus workload on several days was recorded in each group. In most groups, recordings were made during a full working day (excluding lunch break). However, in the early studies, recordings were made only during representative work tasks performed during most of the working day, due to limited recording duration of the equipment. The measurements were thus representative for each occupation.

The data were analysed using EMINGO software, a program developed at our division for the analysis of ElectroMyography, INclinometry and GOniometry. The characteristics of, and interrelations between, the various exposure measures have been evaluated previously [43, 44].

#### Postures and velocities in the head and upper arm

Work postures and movements were recorded using inclinometry, based on triaxial accelerometers (INC logger, Logger Teknologi HB, Åkarp, Sweden). The inclinometers were fixed on the forehead in 148 male workers (17 groups) and 357 female workers (27 groups), and on the outside of the upper arm, below the insertion of the deltoid muscle, in 148 male workers (17 groups) and 362 female workers (25 groups) [19, 48, 49]. The 90th percentile of head forward inclination and right upper arm elevation, and the 50th percentiles of the absolute angular velocity distributions for the head and right upper arm were recorded for each worker.

#### Activity in the trapezius and the forearm extensor muscles

Bipolar surface electromyography (EMG) was used to record the muscular load of the right trapezius muscle in 141 male workers (16 groups) and 506 female workers (29 groups), and of the forearm extensor muscles in 129 male workers (15 groups) and 267 female workers (23 groups) [50, 51]. The data were normalized to the maximal EMG activity (maximal voluntary electric activity: MVE) recorded during maximal voluntary contractions at 90° arm elevation and maximal handgrip, for the trapezius muscle and forearm extensor muscle, respectively. The muscular peak load (90th percentile) in the right forearm extensors and right trapezius muscle was then calculated.

#### Postures and velocities of the wrist

Postures and velocities of the right wrist were measured using flexible biaxial electrogoniometers (Biometrics Ltd., Newport, UK) in 139 male workers (16 groups) and 546 female workers (34 groups) [43, 52, 53]. The 50th percentiles of the angular distributions and the absolute angular velocity distributions for the right side were calculated for each subject [52].

### Outcome assessment

#### Complaints

Complaints (pain or discomfort) in the neck and right shoulder, elbow and hand during the past seven days were recorded for 1105 men and 4727 women using the Nordic Questionnaire (a widely used questionnaire with questions on complaints from different body regions during the past twelve months and past seven days) [54, 55].

#### Diagnoses

Experienced physicians or physiotherapists performed a standardized clinical examination of the neck, and right shoulder, elbow and hand in most of the occupational groups (920 men and 2366 women) [45, 56]. Diagnoses were made according to predefined criteria. Four specific diagnoses are addressed in the present analyses: tension neck syndrome and (right side) rotator cuff syndrome (including supraspinatus tendonitis, infraspinatus tendonitis and bicipital tendonitis), acromioclavicular syndrome and carpal tunnel syndrome.

### Psychosocial work-environment factors

Information on psychosocial working conditions were available for most participants (884/1107 male and 3627/4733 female). In the majority of cases, we used a Swedish version of the Job Content Questionnaire (JCQ), scoring job control, job demands and job support [57–59]. For each item, the participant reported his or her degree of agreement with various statements on a four-point scale. From the results, the mean scores for job demands (seven items), job control (nine items) and job support (eight items) were calculated [60]. Higher scores indicated higher demands, better control and better support (Table 1).

In four groups, psychosocial data were collected by using the Rubenowitz standardized questionnaire [61], and in two with the Copenhagen Psychosocial Questionnaire (COPSOQ) [62]. They cover largely the same aspects as JCQ. For these six groups, data have been transformed into JCQ scores [24] for each participant (Table 1).

### Statistical methods

All the available data on participants were compiled into one large data set. Each participant was then assigned the mean value of physical exposure registered in his or her occupational group. All statistical analyses were carried out using SPSS Statistics, version 22 (IBM Corp.). We regarded *p*-values < 0.05 as indicating statistical significance. Spearman’s rank correlation coefficient (r_S_) was used to assess correlations between assigned physical exposures and reported psychosocial factors. The data for males and females were analysed separately, since there were marked differences between the sexes both in exposure and in the prevalence of musculoskeletal disorders [63]. The associations between each combination of physical exposure and musculoskeletal disorder were modelled separately, using generalized linear regression models. Poisson regression was used to test differences between men and women, and between young and old subjects (dichotomized by median age). The outcome variables (i.e. the occurrence of a musculoskeletal disorder) were assumed to be a Poisson count with log link, and with constant offset of unity.

Prevalence ratios (PRs) with 95% confidence intervals (CIs) were estimated, together with robust estimation of the covariance matrix, in an unadjusted model and a model adjusted for age (divided into five groups: ≤ 31, 32–38, 39–46, 47–53 and ≥ 54 years old). [As sensitivity analyses, the individual factors BMI (continuous), smoking (dichotomous), employment time (continuous), civil status (dichotomous), children at home (dichotomous), exercise (dichotomous) and domestic work (dichotomous) in the age-adjusted models, were included one at a time, although these additional covariates were not available for all occupational groups. A change in PR of 10% was considered a relevant change in the association [64].

When statistically significant associations were found between the physical exposure and the outcome, some multivariate models including velocity and muscular activity were also tested, with adjustment for age. For neck/shoulder complaints and diagnoses, upper arm velocity and trapezius activity were included, while for elbow/hand complaints and carpal tunnel syndrome wrist velocity and forearm extensor activity were included.

Finally, we introduced the three psychosocial variables, treated as continuous data, in extended models. Age-adjusted associations between physical exposure and outcome were determined for the sub-cohorts with complete data sets, and were compared with the associations when psychosocial variables were included.

For a few associations data are shown in figures. The male and female populations were then divided into quintiles, according to physical exposure. The prevalences in these exposure quintiles are plotted at the median exposure within each quintile. Curves describing the associations, calculated by Poisson regression, are also presented. For these associations, tentative values of the attributable fraction among the highest exposed quintile were also calculated using the expression: (prevalence in highest exposure quintile – prevalence in lowest)/prevalence in highest exposure quintile.

## Results

### Physical exposure

Means and ranges of the physical exposure are presented in Table 2. A wide range of exposures was found. For example, upper arm velocity in men varied between 6 and 209 °/s (50th percentile). Some physical exposures were strongly correlated (Table 3).

**Table 2:** Physical exposure on the right side

	Men					Women				
	N_g_	N_w_	N_p_	Mean	(Range)	N_g_	N_w_	N_p_	Mean	(Range)
Head										
Forward inclination (°) 90th percentile	17	148	1107	47	(21–66)	27	357	3848	40	(25–52)
Velocity (°/s) 50th percentile	17	148	1107	16	(3–51)	27	357	3848	11	(4–21)
Trapezius										
Activity (% MVE) 90th percentile	16	141	1051	12	(6–31)	29	506	4233	15	(8–24)
Upper arm										
Elevation (°) 90th percentile	17	148	1107	51	(42–58)	25	362	3828	53	(42–67)
Velocity (°/s) 50th percentile	17	148	1107	45	(6–209)	24	342	3804	31	(6–73)
Forearm extensor										
Activity (% MVE) 90th percentile	15	129	931	21	(3–52)	23	267	3521	25	(12–41)
Wrist										
Palmar flexion (°) 50th percentile	16	139	1051	−11	(−21 – − 2)	34	546	4669	−13	(−30–3)
Velocity (°/s) 50th percentile	16	139	1051	16	(2–55)	34	546	4669	16	(2–45)

Number of groups (N_g_) and workers (N_w_) with recordings. Number of participants that were assigned exposure data (N_p_). Means and ranges of assigned exposure

**Table 3:** Correlation matrix (Spearman’s rank correlation coefficients; r_s_) for assigned physical exposures of the neck and right arm and for the reported psychosocial factors for men (*N* = 765–1113)/women (*N* = 2802–4673)

	Head	Trapezius	Upper arm		Forearm extensors	Wrist		Psychosocial factors		
	Velocity p50	Activity p90	Elevation p90	Velocity p50	Activity p90	Flexion P50	Velocity p50	Job demands	Job control	Job support
Head										
Forward inclination (p90)	0.8/0.3	0.7/0.6	0.5/0.5	0.7/0.2	0.7/0.0	0.6/0.7	0.8/−0.1	− 0.2/0.2	− 0.1/0.2	0.0/0.0
Velocity (p50)		0.9/0.7	0.4/0.6	1.0/1.0	0.8/0.7	0.7/0.7	0.9/0.8	−0.2/0.1	− 0.4/− 0.2	−0.1/− 0.2
Trapezius										
Activity (p90)			0.5/0.6	0.9/0.8	0.8/0.7	0.4/0.7	0.9/0.7	−0.1/0.0	−0.4/− 0.3	−0.1/− 0.2
Upper arm										
Elevation (p90)				0.4/0.5	0.4/0.6	0.5/0.5	0.3/0.4	−0.1/0.1	0.1/0.1	0.2/−0.1
Velocity (p50)					0.8/0.8	0.5/0.6	1.0/0.9	−0.1/0.0	− 0.4/− 0.3	−0.2/− 0.2
Forearm extensors										
Activity (p90)						0.6/0.3	0.9/0.8	−0.2/−0.2	−0.4/− 0.2	−0.1/− 0.2
Wrist										
Palmar flexion (p50)							0.6/0.4	−0.1/0.1	−0.3/− 0.1	0.0/− 0.2
Velocity (p50)								−0.1/0.0	−0.5/− 0.4	−0.2/− 0.3
Psychosocial factors										
Job demands									−0.1/0.0	− 0.1/0.0
Job control										0.5/0.3

For all values above 0.1 *p* < 0.05

Table 1 presents individual characteristics for subjects stratified in quintiles of wrist velocity. For some of these characteristics, there were large differences across occupational groups. For example, smoking was more common in participants with high wrist velocity. Among men, employment time was longer among those with low wrist velocity.

A negative correlation was found between wrist velocity and job control (r_S_ = − 0.4 – -0.5), as can also be seen from Table 1. Other associations between physical exposures and psychosocial factors were weaker.

### Neck/shoulder

#### Complaints

The prevalence of neck/shoulder complaints during the past seven days was higher among women (45%) than in men (30%) (Table 4). The prevalence in men was higher in older than in younger participants. The prevalence of neck/shoulder complaints decreased with increasing forward head inclination, but increased, with increasing trapezius activity, as well as with increasing head, upper arm and wrist velocity, in women (Table 5). Adjustment for age had no significant effect on these associations. None of the other individual factors had any significant influence on the associations between physical exposure and neck/shoulder complaints (not in table).

**Table 4:** Prevalence of musculoskeletal complaints during the past 7 days and diagnoses in the neck and right upper limb

	Men			Women		
	All N (%)	≤41 years N (%)	> 41 years N (%)	N (%)	≤43 years N (%)	> 43 years N (%)
Complaints	*N* = 1105	*N* = 544	*N* = 561	*N* = 4727	*N* = 2325	*N* = 2402
Neck/shoulder	334 (30)^aaa^	145 (27)^b^	189 (34)^b^	2137 (45)^aaa^	1059 (46)	1078 (45)
Elbow/hand	211 (19)^aaa^	99 (18)	112 (20)	1285 (27)^aaa^	508 (22)^bbb^	777 (32)^bbb^
Diagnoses	*N* = 920	*N* = 484	*N* = 436	*N* = 2366	*N* = 1248	*N* = 1118
Tension neck syndrome	50 (5)^aaa^	25 (5)	25 (6)	383 (16)^aaa^	218 (18)	165 (15)
Rotator cuff tendonitis	44 (5)^aa^	11 (2)^bbb^	33 (8) ^bbb^	185 (8)^aa^	85 (7)	100 (9)
Acromioclavicular syndrome	29 (3)^aa^	10 (2)	19 (4)	141 (6)^aa^	55 (4)^bb^	86 (8)^bb^
Carpal tunnel syndrome	18 (2)^a^	11 (2)	7 (2)	87 (4)^a^	36 (3)^b^	51 (5)^b^

^a^Men vs. women, ^a^*p* < 0.05, ^aa^*p* < 0.01, ^aaa^*p* < 0.001

^b^Younger vs. older, ^b^*p* < 0.05, ^bb^*p* < 0.01, ^bbb^*p* < 0.001

Differences between men and women, as well as between younger and older participants, were calculated using Poisson regression

**Table 5:** Associations between physical exposure on the right side, as well as psychosocial factors, and complaints during the past 7 days in the neck and right upper limb, calculated with Poisson regression

	N	Neck/shoulder		Elbow/hand	
		Crude PR (95% CI)	Age-adjusted PR (95% CI)	Crude PR (95% CI)	Age-adjusted PR (95% CI)
Physical exposure					
Head					
Forward inclination (°) 90th percentile					
Men	1105	0.96 (0.89–1.04)	0.96 (0.89–1.04)	**1.22 (1.09–1.37)**	**1.23 (1.09–1.37)**
Women	3844	**0.94 (0.90–0.99)**	**0.94 (0.89–0.98)**	1.03 (0.95–1.11)	0.98 (0.90–1.06)
Velocity (°/s) 50th percentile					
Men	1105	1.00 (0.92–1.09)	1.03 (0.94–1.13)	**1.40 (1.28–1.53)**	**1.42 (1.29–1.56)**
Women	3844	**1.11 (1.03–1.20)**	**1.11 (1.03–1.21)**	**1.63 (1.46–1.82)**	**1.70 (1.53–1.90)**
Trapezius					
Activity (%MVE) 90th percentile					
Men	1049	1.11 (0.90–1.36)	1.17 (0.96–1.43)	**2.10 (1.82–2.43)**	**2.15 (1.84–2.50)**
Women	4227	**1.23 (1.13–1.34)**	**1.24 (1.13–1.35)**	**1.80 (1.59–2.06)**	**1.89 (1.66–2.14)**
Upper arm					
Elevation (°) 90th percentile					
Men	1105	0.93 (0.75–1.15)	0.91 (0.73–1.13)	1.27 (0.92–1.75)	1.27 (0.92–1.75)
Women	3823	0.97 (0.91–1.02)	0.97 (0.91–1.03)	0.93 (0.85–1.02)	0.95 (0.86–1.04)
Velocity (°/s) 50th percentile					
Men	1105	1.01 (0.99–1.03)	1.01 (0.99–1.04)	**1.08 (1.06–1.10)**	**1.09 (1.07–1.11)**
Women	3799	**1.05 (1.03–1.07)**	**1.05 (1.03–1.07)**	**1.13 (1.10–1.16)**	**1.15 (1.12–1.17)**
Forearm extensors					
Activity (%MVE) 90th percentile					
Men	930	0.97 (0.87–1.09)	1.00 (0.89–1.12)	**1.40 (1.26–1.56)**	**1.43 (1.28–1.60)**
Women	3516	1.05 (0.99–1.10)	1.05 (0.99–1.11)	**1.27 (1.17–1.37)**	**1.36 (1.26–1.47)**
Wrist					
Palmar flexion (°) 50th percentile					
Men	1049	0.94 (0.75–1.17)	1.00 (0.79–1.26)	**1.50 (1.09–2.06)**	**1.56 (1.13–2.14)**
Women	4663	0.99 (0.95–1.03)	0.99 (0.95–1.03)	**1.24 (1.15–1.33)**	**1.23 (1.14–1.32)**
Velocity (°/s) 50th percentile					
Men	1049	1.02 (0.95–1.10)	1.05 (0.98–1.13)	**1.35 (1.25–1.45)**	**1.37 (1.27–1.48)**
Women	4663	**1.09 (1.06–1.12)**	**1.09 (1.06–1.13)**	**1.26 (1.21–1.32)**	**1.31 (1.26–1.37)**
Psychosocial factors					
Job demands					
Men	857	**1.29 (1.06–1.58)**	**1.29 (1.06–1.57)**	**1.30 (1.00–1.69)**	**1.31 (1.00–1.71)**
Women	3584	**1.34 (1.24–1.44)**	**1.33 (1.24–1.44)**	**1.43 (1.27–1.60)**	**1.39 (1.25–1.56)**
Job control					
Men	876	0.92 (0.79–1.08)	0.86 (0.74–1.01)	**0.61 (0.51–0.74)**	**0.59 (0.49–0.71)**
Women	3608	**0.80 (0.75–0.85)**	**0.80 (0.75–0.86)**	**0.66 (0.60–0.72)**	**0.61 (0.56–0.66)**
Job support					
Men	875	0.83 (0.68–1.01)	**0.81 (0.67–0.99)**	**0.71 (0.56–0.92)**	**0.70 (0.55–0.90)**
Women	3596	**0.84 (0.79–0.91)**	**0.84 (0.78–0.91)**	**0.70 (0.63–0.78)**	**0.69 (0.62–0.77)**

Prevalence ratio (PR) and 95% confidence interval (CI). Results in bold face are statistically significant. The PRs are expressed per 10°, 10°/s, 10%MVE and for psychosocial factors per step on the 1–4 point scale

When the upper arm velocity and trapezius activity were introduced into an age-adjusted multivariate model for women, only the association between upper arm velocity and the prevalence of neck/shoulder complaints remained statistically significant (prevalence ratio 1.001, 95% confidence interval 1.003–1.010, not in table).

The prevalence of neck/shoulder complaints increased with increasing job demands in both sexes, and also increased with decreasing job control and job support in women (Table 5). Adjustment for psychosocial factors in an extended model reduced the association between trapezius activity and neck/shoulder complaints in women, but the relation was still statistically significant (Table 6).

**Table 6:** Associations between physical exposure on the right side, and complaints during the past 7 days in neck and right upper limb, calculated with Poisson regression

	N	Neck/shoulder		Elbow/hand	
		Age-adjusted PR (95% CI)	Extended model PR (95% CI)	Age-adjusted PR (95% CI)	Extended model PR (95% CI)
Head					
Forward inclination (°) 90th percentile					
Men	847	0.99 (0.90–1.09)	1.01 (0.91–1.11)	**1.31 (1.15**–**1.49)**	**1.30 (1.13–1.49)**
Women	3078	0.97 (0.91–1.04)	0.98 (0.91–1.04)	1.00 (0.90–1.13)	1.07 (0.97–1.18)
Velocity (°/s) 50th percentile					
Men	847	1.04 (0.93–1.16)	1.05 (0.93–1.18)	**1.50 (1.33**–**1.68)**	**1.42 (1.24–1.62)**
Women	3078	**1.18 (1.08**–**1.30)**	1.08 (0.98–1.19)	**1.76 (1.56**–**2.00)**	**1.51 (1.32–1.73)**
Trapezius					
Activity (%MVE) 90th percentile					
Men	847	1.18 (0.91–1.51)	1.16 (0.89–1.50)	**2.40 (2.01**–**2.88)**	**2.06 (1.66–2.55)**
Women	3537	**1.28 (1.16**–**1.41)**	**1.15 (1.04–1.27)**	**2.07 (1.81**–**2.37)**	**1.66 (1.43–1.92)**
Upper arm					
Elevation (°) 90th percentile					
Men	847	0.94 (0.73–1.23)	1.02 (0.77–1.33)	1.40 (0.96–2.04)	**1.56 (1.09–2.23)**
Women	3116	1.00 (0.93–1.08)	0.99 (0.92–1.08)	1.09 (0.98–1.22)	**1.13 (1.01–1.26)**
Velocity (°/s) 50th percentile					
Men	847	1.01 (0.99–1.04)	1.01 (0.98–1.04)	**1.10 (1.08**–**1.13)**	**1.09 (1.06–1.12)**
Women	3116	**1.05 (1.03**–**1.08)**	**1.03 (1.01–1.06)**	**1.16 (1.12**–**1.19)**	**1.11 (1.08–1.15)**
Forearm extensors					
Activity (%MVE) 90th percentile					
Men	753	1.02 (0.88–1.17)	1.05 (0.91–1.23)	**1.68 (1.48**–**1.91)**	**1.62 (1.40–1.88)**
Women	2757	**1.13 (1.06**–**1.20)**	**1.12 (1.04–1.19)**	**1.54 (1.41**–**1.67)**	**1.48 (1.35–1.62)**
Wrist					
Palmar flexion (°) 50th percentile					
Men	847	1.14 (0.86–1.50)	1.18 (0.87–1.59)	**1.94 (1.36**–**2.77)**	**1.65 (1.13–2.40)**
Women	3537	1.02 (0.97–1.08)	0.97 (0.92–1.02)	**1.24 (1.13**–**1.36)**	**1.12 (1.03–1.22)**
Velocity (°/s) 50th percentile					
Men	847	1.05 (0.96–1.15)	1.05 (0.95–1.16)	**1.48 (1.36**–**1.62)**	**1.45 (1.29–1.62)**
Women	3537	**1.11 (1.07**–**1.14)**	**1.06 (1.02**–**1.11)**	**1.32 (1.26**–**1.38)**	**1.22 (1.15–1.29)**

Prevalence ratio (PR) and 95% confidence interval (CI), age-adjusted, and adjusted for age and psychosocial factors (Extended model), for subjects with complete data sets. Results in bold face are statistically significant. The PRs are expressed per 10°, 10°/s, 10%MVE

#### Tension neck syndrome

**Fig. 1:**
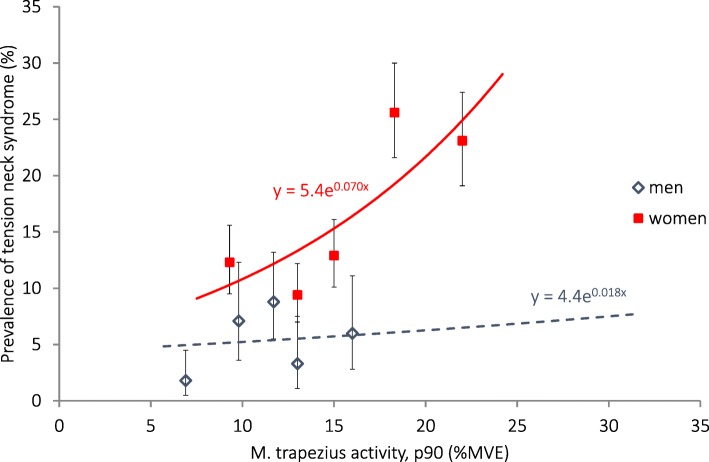
Association between the prevalence of tension neck syndrome and right trapezius muscle activity (90th percentile) calculated with Poisson regression. The symbols denote the prevalences with exact 95% confidence intervals, and the median exposures within sex-specific exposure quintiles in 2366 women and 920 men

Tension neck syndrome was three times more common among women than in men (Table 4), and was evenly distributed among the younger and older participants. The prevalence of this diagnosis in women increased with increasing head and upper arm velocity, and with increasing trapezius activity (Table 7). In the lowest exposure quintile, the median exposure was 9%MVE (Fig. 1) and the prevalence (estimated from the association that was calculated by Poisson regression) was 10%. Corresponding values for the highest quintile were 22%MVE and 25%. Hence, the estimated attributable fraction was 58% among female workers in the highest quintile of trapezius activity. The prevalence increased in both sexes with increasing forearm extensor activity, as well as with increasing wrist palmar flexion and velocity. Adjustment for age had only a minor influence on the associations (not in table). Adjustment for other individual factors had no significant influence on the PRs.

**Table 7:** Associations between physical exposure on the right side, as well as psychosocial factors, and diagnosed disorders in the neck and right upper limb, calculated with Poisson regression

	N	Tension neck syndrome		Rotator cuff tendonitis		Acromioclavicular syndrome		Carpal tunnel syndrome	
		Crude PR (95% CI)	Age-adjusted PR (95% CI)	Crude PR (95% CI)	Age-adjusted PR (95% CI)	Crude PR (95% CI)	Age-adjusted PR (95% CI)	Crude PR (95% CI)	Age-adjusted PR (95% CI)
Physical exposure									
Head									
Inclination (°) p90									
Men	920	1.16 (0.98–1.38)	1.18 (0.99–1.40)	1.07 (0.84–1.37)	1.11 (0.87–1.42)	1.07 (0.86–1.34)	1.09 (0.87–1.37)	**2.41 (1.51–3.86)**	**2.42 (1.50–3.92)**
Women	1825	1.20 (0.98–1.45)	1.16 (0.96–1.41)	0.96 (0.75–1.22)	0.92 (0.72–1.19)	1.09 (0.82–1.43)	0.99 (0.74–1.33)	**2.49 (1.60–3.86**	**2.28 (1.45–3.59)**
Velocity (°/s) p50									
Men	920	1.16 (0.97–1.38)	1.18 (0.98–1.43)	1.24 (0.96–1.60)	**1.40 (1.06–1.85)**	1.26 (0.94–1.69)	1.34 (0.98–1.83)	**2.11 (1.68–2.66)**	**2.30 (1.73–3.06)**
Women	1825	**1.93 (1.51–2.46)**	**1.91 (1.51–2.41)**	**1.77 (1.28–2.46)**	**1.78 (1.28–2.48)**	1.11 (0.73–1.69)	1.14 (0.75–1.72)	**2.24 (1.27–3.95)**	**2.23 (1.30–3.83)**
Trapezius									
(%MVE) p90									
Men	920	1.20 (0.87–1.66)	1.23 (0.88–1.72)	1.49 (0.89–2.49)	**1.81 (1.13–2.90)**	1.69 (0.97–2.93)	**1.90 (1.10–3.27)**	**3.83 (2.60–5.78)**	**4.07 (2.75–6.03)**
Women	2366	**2.00 (1.61–2.49)**	**2.00 (1.61–2.48)**	**1.86 (1.33–2.59)**	**1.87 (1.33–2.61)**	**1.53 (1.04–2.26)**	**1.55 (1.04–2.31)**	**3.99 (2.50–6.36)**	**4.06 (2.54–6.51)**
Upper arm									
Elevation (°) p90									
Men	920	1.37 (0.83–2.25)	1.38 (0.84–2.25)	0.87 (0.44–1.69)	0.86 (0.44–1.69)	1.55 (0.75–3.21)	1.53 (0.74–3.17)	1.53 (0.54–4.31)	1.62 (0.58–4.49)
Women	1878	1.07 (0.89–1.29)	1.09 (0.90–1.31)	1.29 (0.97–1.71)	1.28 (0.96–1.71)	1.05 (0.79–1.39)	1.03 (0.77–1.38)	**1.56 (1.11–2.20)**	**1.56 (1.09–2.24)**
Velocity (°/s) p50									
Men	920	1.03 (0.99–1.07)	1.03 (0.99–1.07)	**1.06 (1.01–1.11)**	**1.09 (1.04–1.15)**	**1.06 (1.00–1.13)**	**1.08 (1.02–1.15)**	**1.17 (1.12–1.22)**	**1.19 (1.13–1.25)**
Women	1878	**1.21 (1.15–1.28)**	**1.20 (1.14–1.26)**	**1.13 (1.05–1.22)**	**1.13 (1.05–1.22)**	1.00 (0.90–1.10)	1.00 (0.91–1.11)	**1.20 (1.07–1.35)**	**1.19 (1.07–1.34)**
Forearm extensors									
(%MVE) p90									
Men	800	**1.32 (1.09–1.61)**	**1.38 (1.10–1.73)**	**1.34 (1.01–1.78)**	**1.58 (1.12–2.23)**	**1.48 (1.09–2.00)**	**1.63 (1.17–2.28)**	**1.93 (1.43–2.56)**	**2.09 (1.38–3.16)**
Women	1437	**1.34 (1.13–1.58)**	**1.32 (1.12–1.56)**	**1.34 (1.08–1.66)**	**1.36 (1.10–1.69)**	0.94 (0.70–1.27)	0.98 (0.73–1.33)	**1.56 (1.07–2.29)**	**1.64 (1.15–2.36)**
Wrist									
Flexion (°) p50									
Men	920	**4.51 (2.31–8.84)**	**4.91 (2.49–9.67)**	1.49 (0.72–3.08)	1.75 (0.85–3.61)	**3.66 (1.49–9.01)**	**3.94 (1.62–9.58)**	**3.44 (1.60–7.37)**	**3.50 (1.53–7.97)**
Women	2366	**1.14 (1.01–1.28)**	**1.15 (1.02–1.29)**	**1.37 (1.14–1.64)**	**1.36 (1.13–1.64)**	**1.28 (1.03–1.61)**	**1.28 (1.02–1.60)**	**1.99 (1.50–2.65)**	**2.01 (1.49–2.71)**
Velocity (°/s) p50									
Men	920	**1.20 (1.05–1.38)**	**1.23 (1.05–1.43)**	**1.27 (1.05–1.55)**	**1.45 (1.17–1.78)**	**1.31 (1.05–1.63)**	**1.41 (1.12–1.78)**	**2.10 (1.72–2.56)**	**2.35 (1.78–3.10)**
Women	2366	**1.29 (1.20–1.38)**	**1.30 (1.21–1.40)**	**1.34 (1.20–1.50)**	**1.37 (1.22–1.53)**	**1.21 (1.04–1.41)**	**1.25 (1.08–1.45)**	**1.43 (1.20–1.70)**	**1.49 (1.26–1.76)**
Psychosocial factors									
Job demands									
Men	735	0.93 (0.48–1.79)	0.93 (0.49–1.77)	**1.74 (1.03–2.95)**	**1.84 (1.10–3.07)**	**2.24 (1.12–4.49)**	**2.26 (1.14–4.45)**	**3.18 (1.83–5.51)**	**3.49 (1.96–6.20)**
Women	1878	**1.53 (1.24–1.88)**	**1.52 (1.24–1.87)**	**1.47 (1.09–1.99)**	**1.48 (1.09–2.00)**	**1.86 (1.33–2.59)**	**1.91 (1.36–2.67)**	**1.59 (1.01–2.48)**	**1.60 (1.03–2.51)**
Job control									
Men	754	0.70 (0.49–1.02)	**0.65 (0.45–0.94)**	0.78 (0.49–1.25)	0.65 (0.42–1.03)	0.69 (0.42–1.15)	0.66 (0.41–1.08)	**0.30 (0.13–0.68)**	**0.30 (0.14–0.66)**
Women	1897	**0.55 (0.47–0.65)**	**0.55 (0.46–0.65)**	**0.58 (0.45–0.75)**	**0.55 (0.42–0.72)**	**0.68 (0.52–0.89)**	**0.64 (0.48–0.84)**	**0.55 (0.40–0.76)**	**0.49 (0.35–0.68)**
Job support									
Men	753	0.64 (0.33–1.25)	0.59 (0.31–1.11)	0.51 (0.26–1.03)	**0.43 (0.24–0.78)**	0.55 (0.25–1.24)	0.53 (0.24–1.15)	**0.26 (0.11–0.58)**	**0.23 (0.09–0.55)**
Women	1884	**0.68 (0.55–0.83)**	**0.68 (0.55–0.83)**	**0.68 (0.51–0.91)**	**0.67 (0.50–0.90)**	**0.61 (0.44–0.83)**	**0.59 (0.43–0.81)**	**0.46 (0.29–0.74)**	**0.43 (0.27–0.71)**

Prevalence ratio (PR) and 95% confidence interval (CI). Results in bold face are statistically significant. The PRs are expressed per 10°, 10°/s, 10%MVE and for psychosocial factors per step on the 1–4 point scale

As for neck/shoulder complaints, only the association between upper arm velocity and the prevalence of tension neck syndrome was statistically significant in the multivariate model that also included trapezius activity (prevalence ratio 1.02, 95% confidence interval 1.01–1.03, not in table).

The prevalence of tension neck syndrome in women was significantly associated with increasing job demands and decreasing job control and job support (Table 7). Adjustment for psychosocial factors attenuated the association between wrist palmar flexion and tension neck syndrome in women, and it was no longer statistically significant (Table 8); all other associations remained.

**Table 8:** Associations between physical exposure on the right side, and diagnosed disorders in neck and dominant upper limb, calculated with Poisson regression

N		Tension neck syndrome		Rotator cuff tendonitis		Acromioclavicular syndrome		Carpal tunnel syndrome	
		Age-adjusted PR (95% CI)	Extended model PR (95% CI)	Age-adjusted PR (95% CI)	Extended model PR (95% CI)	Age-adjusted PR (95% CI)	Extended model PR (95% CI)	Age-adjusted PR (95% CI)	Extended model PR (95% CI)
Head									
Inclination (°) p90									
Men	726	**1.30 (1.04–1.64)**	1.27 (0.99–1.63)	1.14 (0.84–1.56)	1.19 (0.85–1.67)	1.15 (0.86–1.54)	1.22 (0.88–1.69)	**2.09 (1.20**–**3.62**	**2.14 (1.16–3.93)**
Women	1381	1.33 (0.99–1.78)	1.22 (0.95–1.57)	0.94 (0.67–1.32)	0.94 (0.69–1.29)	0.92 (0.64–1.35)	0.82 (0.58–1.17)	**1.72 (1.04**–**2.85)**	1.60 (0.93–2.76)
Velocity (°/s) p50									
Men	726	**1.33 (1.04–1.71)**	1.26 (0.94–1.69)	1.38 (0.99–1.93)	1.35 (0.92–1.99)	**1.52 (1.04–2.22)**	**1.58 (1.04–2.39)**	**2.24 (1.57–3.19)**	**1.74 (1.17–2.58)**
Women	1381	**2.50 (1.87–3.33)**	**1.97 (1.45–2.69)**	**1.68 (1.14**–**2.49)**	1.41 (0.93–2.14)	1.08 (0.66–1.78)	0.89 (0.54–1.45)	**2.05 (1.16**–**3.63)**	1.75 (0.83–3.72)
Trapezius									
(%MVE) p90									
Men	726	1.49 (1.00–2.23)	1.27 (0.79–2.04)	1.55 (0.86–2.78)	1.22 (0.62–2.38)	**2.38 (1.35**–**4.21)**	**2.21 (1.20–4.09)**	**3.03 (2.02**–**4.58)**	**1.76 (1.12–2.78)**
Women	1840	**2.41 (1.86–3.10)**	**1.83 (1.35–2.47)**	**2.15 (1.46**–**3.17)**	**1.60 (1.04–2.46)**	**1.60 (1.01**–**2.53)**	1.10 (0.68–1.78)	**3.84 (2.17**–**6.80)**	**2.56 (1.33–4.93)**
Upper arm									
Elevation (°) p90									
Men	726	**2.13 (1.18–3.84)**	**2.21 (1.27–3.87)**	0.67 (0.34–1.33)	0.85 (0.44–1.64)	1.70 (0.73–3.95)	2.08 (0.89–4.81)	1.02 (0.39–2.65)	1.26 (0.58–2.76)
Women	1419	**1.30 (1.02–1.65)**	1.29 (0.98–1.69)	1.39 (0.97–1.98)	**1.52 (1.04–2.23)**	1.10 (0.76–1.60)	1.04 (0.67–1.62)	1.05 (0.64 – 1.72)	1.07 (0.60–1.92)
Velocity (°/s) p50									
Men	726	**1.06 (1.01–1.11)**	1.05 (0.98–1.11)	**1.09 (1.03**–**1.16)**	1.07 (0.99–1.17)	**1.11 (1.04**–**1.19)**	**1.12 (1.03–1.22)**	**1.17 (1.11**–**1.24)**	**1.10 (1.03–1.19)**
Women	1419	**1.27 (1.19–1.35)**	**1.20 (1.12–1.29)**	**1.13** (**1.03**–**1.23)**	1.07 (0.98–1.17)	0.99 (0.88 – 1.11)	0.94 (0.83–1.05)	**1.17 (1.03**–**1.34)**	1.13 (0.95–1.34)
Forearm extensors									
(%MVE) p90									
Men	631	**1.69 (1.28 – 2.23)**	**1.68 (1.23–2.29)**	1.52 (0.97–2.39)	1.52 (0.93–2.49)	**2.03 (1.46**–**2.82)**	**2.13 (1.45–3.11)**	**2.29 (1.32**–**3.95)**	**1.82 (1.06–3.12)**
Women	1060	**1.41 (1.14–1.75)**	**1.27 (1.01–1.59)**	1.22 (0.93–1.60)	1.15 (0.88–1.50)	0.81 (0.55 – 1.20)	0.83 (0.56–1.23)	1.26 (0.80–2.00)	1.29 (0.78–2.13)
Wrist									
Flexion (°) p50									
Men	726	**5.83 (2.60–13.1)**	**6.51 (2.69–15.8)**	1.88 (0.78–4.52)	2.07 (0.84–5.09)	**4.04 (1.50**–**10.9)**	**4.55 (1.62–12.8)**	**4.30 (1.79**–**10.3)**	**3.55 (1.29–9.79)**
Women	1840	**1.19 (1.04–1.36)**	1.04 (0.91–1.19)	**1.40 (1.13**–**1.74)**	**1.25 (1.01–1.55)**	1.30 (1.00–1.67**)**	1.10 (0.86–1.40)	**2.15 (1.48**–**3.13)**	**1.69 (1.17–2.44)**
Velocity (°/s) p50									
Men	726	**1.35 (1.12–1.64)**	**1.34 (1.02–1.75)**	**1.54 (1.20**–**1.98)**	**1.53 (1.04–2.26)**	**1.61 (1.23**–**2.11)**	**1.77 (1.21–2.57)**	**2.31 (1.67**–**3.21)**	**1.96 (1.23–3.13)**
Women	1840	**1.34 (1.24–1.45)**	**1.21 (1.09–1.35)**	**1.36 (1.20**–**1.54)**	**1.23 (1.05–1.45)**	**1.27 (1.08**–**1.50)**	1.13 (0.93–1.36)	**1.56 (1.29**–**1.89)**	**1.33 (1.04–1.71)**

Prevalence ratio (PR) and 95% confidence interval (CI), age-adjusted, and adjusted for age and psychosocial factors (Extended model), for subjects with complete data sets. Results in bold face are statistically significant. The PRs are expressed per 10°, 10°/s, and 10%MVE

#### Rotator cuff tendonitis

**Fig. 2:**
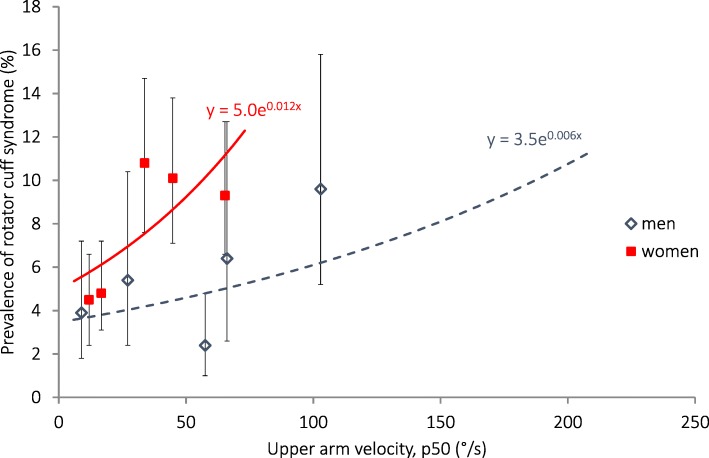
Association between the prevalence of rotation cuff tendinitis on the right side and right upper arm velocity (50th percentile) calculated with Poisson regression. The symbols denote the prevalences with exact 95% confidence intervals, and the median exposures within sex-specific exposure quintiles in 1878 women and 920 men

Rotator cuff tendonitis was also more common among women than in men (Table 4). In men, it was more common among older than among younger participants. Increasing head, upper arm and wrist velocities, trapezius and forearm extensor activities and, in women only, increasing wrist palmar flexion, were associated with increasing prevalence of rotator cuff tendonitis (Table 7). In the highest upper arm velocity quintiles (median 103 °/s in men, 65 °/s in women, Fig. 2), the prevalence that was estimated with Poisson regression was 6% in men and 11% in women. The estimated attributable fraction in men was 41% and in women 48%. The PRs for age-adjusted data were in general only marginally higher than for the unadjusted data. When adjustments were made for other individual factors, BMI reduced the PR for the association between rotator cuff tendonitis and head velocity in women from 2.3 (95% CI 1.4–3.6; not shown) to 1.9 (1.3–3.2). The PR was slightly attenuated in the association with trapezius activity, from 2.3 (1.3–4.0) to 2.0 (1.1–3.4). No such effect was seen in men, and no other adjustments had any significant effect on PR.

Upper arm velocity, but not trapezius activity, was statistically significantly associated with the prevalence of rotator cuff tendonitis in both sexes, in an age-adjusted multivariate model (not in table).

Increasing job demands, decreasing job control (only in women), and decreasing job support were also associated with increasing prevalence of rotator cuff tendonitis. Adjustment for psychosocial factors led to a decrease in most PRs. In women, the association with head velocity, and in men the association with trapezius activity, were reduced such that they were no longer statistically significant (Table 8). Other associations remained statistically significant or were attenuated by less than 10%.

#### Acromioclavicular syndrome

Acromioclavicular syndrome was twice as common in women as in men (Table 4), and was more prevalent among older women than younger women. The prevalence increased in both sexes with increasing trapezius activity, wrist palmar flexion and wrist velocity (Table 7). In men, acromioclavicular syndrome was also positively associated with upper arm velocity and forearm extensor activity, and the associations were somewhat higher after age adjustment. Among men, smoking reduced the PR for the association between wrist flexion and the prevalence of acromioclavicular syndrome, from 8.3 (95% CI 1.8–39; not in table) to 7.3 (1.6–34) among the 677 participants with complete data sets for these variables. Among women, none of the statistically significant associations were influenced by adjustment for other individual factors.

In the age-adjusted multivariate models, upper arm velocity, but not trapezius activity, was statistically significantly associated with the prevalence of acromioclavicular syndrome, in both sexes.

Acromioclavicular syndrome was positively associated with job demands, and, in women, negatively associated with job control and job support (Table 7). In women, adjustment for psychosocial factors attenuated the associations with trapezius activity, wrist palmar flexion and wrist velocity, such that they were no longer statistically significant (Table 8).

### Elbow/hand

#### Complaints

**Fig. 3:**
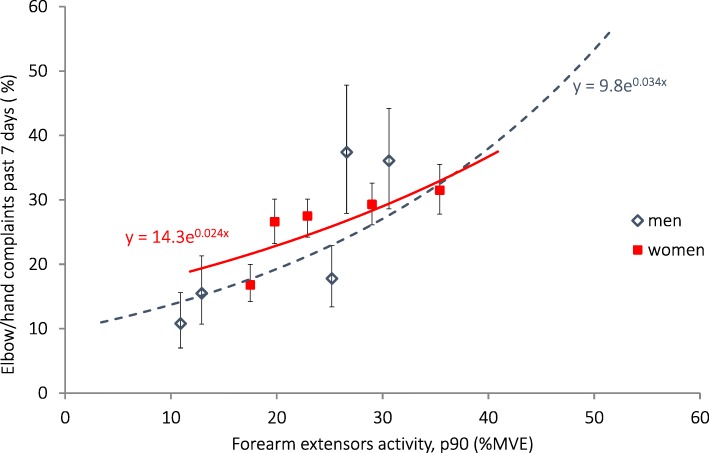
Association between the prevalence of elbow/hand complaints on the right side and right-side extensor muscles activity (90th percentile) calculated with Poisson regression. The symbols denote the prevalences with exact 95% confidence intervals, and the median exposures within sex-specific exposure quintiles in 3516 women and 930 men

The prevalence of elbow/hand complaints was more common among women than in men, and in women more common among older than among younger subjects (Table 4). All physical exposures except upper arm elevation and head inclination (in women), showed significant positive associations with elbow/hand complaints in both sexes (Table 5). In the highest quintiles of forearm extensor activity (median 31% MVE in men, 35%MVE in women, Fig. 3), the prevalence that was estimated with Poisson regression was 28% in men and 33% in women. The estimated attributable fractions were 49% among men and 34% among women. In men, adjustment for smoking reduced the PR for the association between wrist palmar flexion and the prevalence of elbow/hand complaints, from 2.0 (95% CI 1.4–2.9; not in table) to 1.8 (1.2–2.6). None of the other individual characteristics had any significant influence on these associations.

When wrist velocity and forearm extensor activity were introduced into the age-adjusted multivariate models, only wrist velocity was statistically significantly associated with the prevalence of elbow/hand complaints, in both sexes (not in table).

Furthermore, all three aspects of psychosocial environment were associated with elbow/hand complaints (increasing with increasing job demands, decreasing job control and decreasing job support) in both sexes. All associations between physical factors and elbow/hand complaints remained statistically significant after adjustment for psychosocial factors (Table 6).

#### Carpal tunnel syndrome

**Fig. 4:**
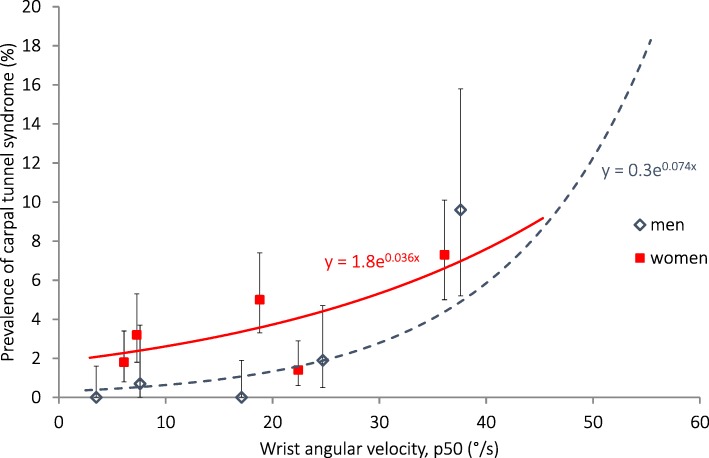
Association between the prevalence of carpal tunnel syndrome on the right side and angular velocity of the right wrist (50th percentile) calculated with Poisson regression. The symbols denote the prevalence with exact 95% confidence intervals, and the median exposures within sex-specific exposure quintiles in 2366 women and 920 men

The prevalence of carpal tunnel syndrome was 4% in women, and more common among older than among younger women (Table 4). It was less common in men, and did not vary with age. It was positively associated with all measures of physical exposure, except upper arm elevation in men (Table 7). In the highest wrist velocity quintiles (38 °/s in men, 36 °/s in women, Fig. 4) the prevalence that was estimated with Poisson regression was 5% in men and 7% in women. The estimated attributable fractions were 92% in men and 66% in women. In general, adjustment for age gave somewhat higher PRs.

In women, several associations were influenced by adjustment for BMI. The association between the prevalence of carpal tunnel syndrome and head velocity was attenuated from 1.5 (95% CI 0.6–3.5; not in table) to 1.2 (0.5–3.0) in subjects with complete data sets for these variables. For trapezius activity, it was attenuated from 4.5 (2.5–8.3) to 3.7 (2.1–6.8). Finally, for wrist palmar flexion it was attenuated from 2.2 (1.4–3.6) to 2.0 (1.3–3.2). No such effect of BMI was seen in men.

Concerning the multivariate models, in men, only wrist velocity was associated with the prevalence of carpal tunnel syndrome. In women, only forearm extensor activity remained in the model.

All three dimensions of the psychosocial work environment were also associated with carpal tunnel syndrome, in both sexes (Table 7). Adjustment for psychosocial factors attenuated the association between head velocity and carpal tunnel syndrome in women by 15%, and it was not statistically significant (Table 8). All other associations remained.

## Discussion

The study confirmed that all the evaluated physical exposures were positively associated with complaints and/or diagnoses in the neck and/or upper extremity. The associations remained after adjustment for age, and in most cases also after adjustment for other individual factors. Importantly, it also remained after adjustment for psychosocial conditions.

The reported attributable fractions in the highest quintiles were mostly around 50%. However, for carpal tunnel syndrome, the value was as high as 92% for the highest quintile of wrist velocity in men.

### Methodological aspects

Compared to our earlier studies, the extended material enabled a more reliable estimate of the exposure-response curves in particular, and the important low-range pattern of the curves is now far better defined. Importantly, the present data set ensures a higher statistical power. Fourteen “new” statistically significant associations were revealed, while six “disappeared”, but without a consistent pattern.

The strengths of this study are that the same methods of assessing both the physical exposures and the outcomes were used in large numbers of occupations and workers, over wide ranges of exposures, which allowed detailed analysis of the exposure-response relationships. A weakness is that complete data sets were not available for all occupations/workers, and several of the analyses could therefore only be carried out on part of the data-set. Nonetheless, most associations were robust to adjustment for individual factors.

Since the study groups were examined cross-sectionally, they are subject to the usual limitations regarding the conclusions on causality. However, overestimation of the risk is unlikely, since subjects with certain disorders would probably avoid work tasks with heavy workloads. Rather, healthy-worker selection might be a problem, causing underestimation of the prevalence of an exposure-induced disorder at long employment times, due to the lack of “unhealthy” workers as a result of high exposure.

Using a large amount of data is likely to limit the bias arising from the use of group-level exposure measures in the exposure-response curves [65]. Although the numbers of measurements of physical exposure are high, we only had sufficient resources to perform demanding recordings on a limited number of subjects in each occupation, and only for one day each. Replacing individual exposure values with group averages does not introduce a bias per se, but rather decreases the statistical precision [66]. However, errors in the estimated group-level averages introduce bias towards the null, given that these estimation errors are independent of the true group-level exposure.

We used information concerning individual characteristics and outcomes. The use of group means for physical exposure may cause a clustering effect, if there are differences in other factors between the groups, which would then be falsely attributed to the exposure. However, we believe this potential problem to be limited. For example, adjustment for age and psychosocial factors caused little change in the slopes. The same was seen after adjustments for BMI and smoking, which, in most cases, did not significantly affect the slopes, in spite of the fact that they have previously been assumed to be risk factors [28, 67–72].

We assessed the psychosocial work environment through questionnaires, which means a considerable risk of reporting bias [73] and the risk of reversed causality for these factors. Furthermore, since they are correlated with the physical risk factors, there will be a risk of over-adjustment if the perceived psychosocial environment is a consequence of the physical environment. Both of these circumstances lead to the risk of underestimation of a true effect of the physical factors when psychosocial and physical factors are introduced into the model.

In summary, we do not believe that our reported associations are systematically overestimated, rather that they might be somewhat biased towards the null.

### Theoretical and practical importance

Our quantitative exposure-response data can be useful in several ways. When musculoskeletal risks are suspected due to the physical working environment, the measurements and exposure-response relationships described in this study can be used to evaluate the predicted impact on risk, long before it has become manifest as health problems. Alternative ways of performing the same work task can then be measured and compared, in order to minimize the predicted risk. Moreover, our data could also be used to establish threshold limit values for physical exposures of the musculoskeletal system, in a way only occasionally [16] done for musculoskeletal workload, but successfully employed for many years regarding chemical, physical and biological risk factors.

The fact that several different physical exposures show exposure-response relationships with disorders in the neck and upper limb is an important clue for causal inference. In the neck/shoulder region, forward head inclination was associated with rotator cuff tendonitis in women. Surprisingly, it was found to be negatively associated with neck complaints, despite the fact that previous studies have found a positive relationship [74]. Indeed, working with 20° neck flexion for more than 40% of the working day has been shown to be a risk factor for sick leave due to neck pain in a prospective cohort study [75]. We found that head velocity also was associated neck complaints, tension neck syndrome and rotator cuff tendonitis in women, which, to the best of our knowledge, has not reported before.

Trapezius activity was also associated with neck/shoulder complaints and tension neck syndrome in women, and with rotator cuff syndrome and acromioclavicular syndrome in both sexes. Tension neck syndrome is a myofascial pain syndrome with tenderness and tightness of the muscles around the neck, which makes the association most credible. When introducing upper arm velocity in multivariate models, trapezius activity no longer showed a statistically significant association with any of the diagnoses. However, as the correlation between upper arm elevation and trapezius activity was as high as 0.8 in women, it is not surprising that we were not able to separate the effects of these. Exposure-response relationships between tension neck syndrome and trapezius activity (and for forearm extensor muscle activities) have not been reported previously, in spite of the long-term interest in EMG as a risk indicator [76].

We found no associations between upper arm elevation and any of the neck/shoulder outcomes, in spite of the fact that it has been reported repeatedly that such exposure is a risk factor for shoulder disorders [10–12, 72]. This is probably because of the fact that, although we have recorded exposures in a wide range of occupations, few of them involved work above shoulder level for any length of time.

Upper arm velocity, on the other hand, was associated with all neck/shoulder outcomes. This reflects repetition in arm movements, which is a risk factor for tension neck syndrome [77]. Interestingly, Dalbøge et al. [78] have shown that an upper arm velocity below 45 °/s (median over the working day) seems to be a safe level regarding the risk of subacromial impingement syndrome. Thus, high velocities of the upper arm throughout the working day, whether repetitive or not, should be avoided. We suggest a threshold limit value of 60 °/s (median over 8-h working day), which should protect workers in occupations with the highest velocities that we have recorded.

In accordance with earlier studies [41, 79], we found strong positive associations between wrist palmar flexion and wrist velocity, and both carpal tunnel syndrome and elbow/hand complaints. Carpal tunnel syndrome is rather uncommon in men; and almost all cases in the highest quintile could be attributed to high wrist velocity. It is obvious from Fig. 4 that a wrist velocity above 20 °/s is undesirable. Carpal tunnel syndrome was also associated with forearm extensor activity, and when introducing this and wrist velocity into the same multivariate model, wrist velocity remained statistically significant in men, while forearm extensor activity remained statistically significant in women. As for the trapezius activity and upper arm velocity mentioned above, this was expected, due to the close correlation between these exposure measures. Still, we interpret the fact that the results differed between men and women to mean that they are both of importance, which is in line with a recently published study by Kapellusch et al. [35].

Finally, several associations were found between forearm and wrist activity, and diagnoses in the neck/shoulders. This has been noted previously [80], and is probably due to the close correlation between activities in the wrist, shoulder and neck. For example, in repetitive industrial work, not only the hands and arms are active, but also the trapezius to stabilize the shoulder, and the neck is bent enabling the worker to see what he or she is doing. This means that wrist electrogoniometry may be used to assess not only the risk of elbow/hand disorders, but also the risks of disorders of the neck/shoulder. We suggest a threshold limit value of 20 °/s (median over 8-h working day).

As pointed out above, muscular activity and the velocity of movements of the head, upper arm or wrist are physical exposures that are very difficult to assess through observations or self-reporting. The fact that they are associated with several of the evaluated disorders underlines the need for technical measurements of the physical exposure when evaluating the ergonomic risk. As technical developments are taking place towards wearable devices for recording postures and movements, we foresee that employers and workers themselves, as well as ergonomists and labour inspectorates, will be able to measure exposure easily over several days in the near future. The results obtained can then be used to predict risks and for comparison with threshold limit values.

## Conclusions

Using an extensive data-set, we found a series of quantitative exposure-response relationships for occupational physical exposures of the neck and upper extremity and disorders of the neck, shoulders and hands. This is in accordance with our earlier studies. The associations were, in general, robust to adjustment for age and other individual factors, as well as for psychosocial conditions. Based on this knowledge we suggest threshold limit values for the upper arm velocity of 60 °/s and for wrist velocity of 20 °/s, as median values over an eight-hour working day.

## Abbreviations

BMI: Body mass index
CI: Confidence interval
COPSOQ: Copenhagen Psychosocial Questionnaire
EMG: Electromyography
JCQ: Job Content Questionnaire
PR: Prevalence ratio
r_S_: Spearman’s rank correlation coefficient
